# Predictors of mortality in COVID-19 patients at Kinshasa University Hospital, Democratic Republic of the Congo, from March to June 2020

**DOI:** 10.11604/pamj.2020.37.105.25279

**Published:** 2020-10-01

**Authors:** Ben Izizag Bepouka, Madone Mandina, Jean Robert Makulo, Murielle Longokolo, Ossam Odio, Nadine Mayasi, Tresor Pata, Godelive Nsangana, Felly Tshikangu, Donatien Mangala, Dupont Maheshe, Serge Nkarnkwin, Jonathan Muamba, Gorby Ndaie, Rodrigue Ngwizani, Yves Yanga, Aliocha Nkodila, Hervé Keke, Yamin Kokusa, Francois Lepira, Innocent Kashongwe, Marcel Mbula, Jean Marie Kayembe, Hippolyte Situakibanza

**Affiliations:** 1 Unit of Infectious Diseases, Kinshasa University Hospital, Kinshasa, Democratic Republic of the Congo,; 2 Unit of Nephrology, Kinshasa University Hospital, Kinshasa, Democratic Republic of the Congo,; 3 Unit of Reanimation, Kinshasa University Hospital, Kinshasa, Democratic Republic of the Congo,; 4 Unit of Vaccinology, World Health Organization, Kinshasa, Democratic Republic of the Congo,; 5 Department of Epidemiology, Ministry of Health, Kinshasa, Democratic Republic of the Congo,; 6 Unit of Pneumology, Kinshasa University Hospital, Kinshasa, Democratic Republic of the Congo

**Keywords:** Survival, mortality, COVID-19, Democratic Republic of the Congo

## Abstract

**Introduction:**

since the 1^st^ case of coronavirus disease 2019 (COVID-19) in Kinshasa on March 10^th^2020, mortality risk factors have not yet been reported. The objectives of the present study were to assess survival and to identify predictors of mortality in COVID-19 patients at Kinshasa University Hospital.

**Methods:**

a retrospective cohort study was conducted, 141 COVID-19 patients admitted at the Kinshasa University Hospital from March 23 to June 15, 2020 were included in the study. Kaplan Meier's method was used to described survival. Predictors of mortality were identified by COX regression models.

**Results:**

of the 141 patients admitted with COVID-19, 67.4 % were men (sex ratio 2H: 1F); their average age was 49.6±16.5 years. The mortality rate in hospitalized patients with COVID-19 was 29% during the study period with 70% deceased within 24 hours of admission. Survival was decreased with the presence of hypertension, diabetes mellitus, low blood oxygen saturation (BOS), severe or critical stage disease. In multivariate analysis, age between 40 and 59 years [adjusted Hazard Ratio (aHR): 4.07; 95% CI: 1.16 - 8.30], age at least 60 years (aHR: 6.65; 95% CI: 1.48-8.88), severe or critical COVID-19 (aHR: 14.05; 95% CI: 6.3-15.67) and presence of dyspnea (aHR: 5.67; 95% CI: 1.46-21.98) were independently and significantly associated with the risk of death.

**Conclusion:**

older age, severe or critical COVID-19 and dyspnea on admission were potential predictors of mortality in patients with COVID-19. These predictors may help clinicians identify patients with a poor prognosis.

## Introduction

In late December 2019, the first case of coronavirus disease 2019 (COVID-19) was identified in people with pneumonia that had been associated with a seafood and live animal market in the city of Wuhan in Hubei, China [1]. As of February 16^th^, 2020, 68,694 confirmed cases of COVID-19 infection had been reported in China, with 1,667 deaths [2]. Although the mortality rate for COVID-19 is only 2.4% lower than for SARS, the number of deaths is much higher due to the high number of infected persons [3]. The World Health Organization (WHO) considers this epidemic to be a public health emergency of international concern [4]. From December 31^st^ to May 9^th^, 2020, there were 3,898,658 cases of COVID-19 with 274,290 deaths worldwide. Africa has 57,860 cases of COVID-19 with 2155 deaths as of May 9^th^, 2020. In the Democratic Republic of the Congo (DRC), from March 10^th^ to May 9^th^, 2020, the country had 991 cases with 41 deaths, i.e. a lethality rate of 4% [2]. According to risk factor for mortality, analysis of data collected by the WHO between 2012 and 2018 identified risk factors for coronavirus; men appeared to be more affected than women, as well as age over 30 years and the presence of co-morbidities [5]. In addition, the association between age, sex and mortality rates of co-morbidities such as hypertension, diabetes, cardiovascular disease, chronic respiratory disease and cancer was identified by various studies [6-9]. Fei Zhou *et al*. identified as factors associated with mortality: advanced age, high Sequential Organ Failure Assessment (SOFA) score and high d-dimer among patients hospitalized in Wuhan from 29^th^ December 2019 to 31^st^ January 2020 [10].

Du RH *et al*. identified four risk factors for COVID-19 death: age ≥65 years, pre-existing concurrent cardiovascular or cerebrovascular diseases, CD3+CD8+ T-cells ≤75 cells: μL-1 and cardiac troponin I ≥0.05 ng/mL-1. The latter two factors, especially, were predictors for mortality of COVID-19 pneumonia patients [11]. In a cohort study using national primary care electronic health record data linked to in-hospital COVID-19 death data, people from Asian and African descents had a substantially higher risk of death from COVID-19, only partially attributable to co-morbidity, deprivation or other risk factors. Deprivation is also a major risk factor for COVID-19 death, which was only partly attributable to co-morbidity or other risk factors [12]. In New York City, older age, chronic cardiac disease, chronic pulmonary disease, higher concentrations of interleukin-6 and higher concentrations of D-dimer were independently associated with in-hospital mortality [13]. In a study in Brazil, Soares *et al*. found that for death outcome in hospitalized patients, only older age and shortness of breath increased the risk [14]. Although the mortality in china was low, the figures in Democratic Republic of the Congo (DRC) seem higher (around 40 as of May 9^th^, 2020) than those of other African countries, although the co-morbidities and factors associated with severe forms may be the same. To our knowledge, the factors associated with mortality are not well known in Africa, hence the need for more studies to understand factors associated with COVID-19 mortality risks in the African context. The objectives of this study were to assess survival and identify predictive factors of mortality from COVID-19 in COVID-19 patients admitted at the Kinshasa University Hospital, one of the largest referral hospitals of the Democratic Republic of Congo.

## Methods

**Study design and period:** the present study was a retrospective cohort covering the period from March 23^rd^ to June 15^th^, 2020.

**Study site:** this study was conducted at the Kinshasa University Hospital, a large regional hospital in Kinshasa, DRC. Kinshasa University Hospital is the tertiary level hospital in the city of Kinshasa with a capacity of 1000 beds. Within the hospital, a COVID-19 treatment center is installed for the management of COVID-19 cases, with an emergency department, a standard hospitalization and a reanimation department where severe or critical cases of COVID-19 are treated. The COVID-19 center also has the capacity to perform emergency hemodialysis, interventional endoscopies and chemotherapy for cancer patients with COVID-19. According to the organization of the COVID-19 response in the DRC, at the provincial level, the management of COVID-19 patients is in COVID-19 treatment centers and health zones. The management of asymptomatic patients is provided by the health zone and depending on the degree of aggravation following a fragility score, patients could be sent to the COVID-19 treatment centers. Mild, moderate, severe or critical patients are managed at the COVID-19 treatment centers. The city of Kinshasa has 14 COVID-19 treatment centers and 35 health zones. As a last resort, all severe or critical cases that cannot be managed in the other treatment centers are referred to the COVID-19 treatment center of KUH, as well as all COVID-19 patients requiring dialysis in Kinshasa. The other provinces of central and western DRC can also refer cases of COVID-19. The COVID-19 treatment center of KUH refers to the health zone or other primary level COVID-19 treatment centers all asymptomatic or mild cases without life-threatening co-morbidities.

**Study population:** this study enrolled all patients who consulted with confirmed COVID-19. COVID-19 patients diagnosed according to World Health Organization (WHO) interim guideline [15].

**Inclusion criteria:** included in this study were patients who were hospitalized as confirmed case of COVID-19. Transferred or escaped patients have not been included.

**Sampling:** the sampling for this study was exhaustive and consecutive. Data collection: each hospitalized patient meeting the inclusion criteria was systematically included in the survey. We obtained from patients´ medical record: sociodemographic, clinical, biological characteristics, co-morbidities and treatment according to the COVID-19 national treatment policy.

### Study definitions

**Mild COVID-19:** any COVID-19 patients with few or no symptoms, temperature <38.5°C, normal respiratory rate (between 12-20 breaths per minute), normal lung auscultation, normal chest X-ray (if available), blood oxygen saturation ≥95%.

**Moderate COVID-19:** any COVID-19 patients with these criteria: temperature ≥38.5°C, respiratory rate between 20 and 30 breaths per minute, normal lung auscultation, normal chest X-ray (if available), blood oxygen saturation between 90 and 95%.

**Severe COVID-19:** any COVID-19 patients with these criteria: temperature ≥38.5°C, respiratory rate ≥30 breaths per minute, signs of severe pneumonia: respiratory rate >30 breaths per minute; signs of severe respiratory distress (nasal flaring, cyanosis, speech difficulty, restlessness, loss of consciousness, chest indrawing); or blood oxygen <90%, pathological chest x-ray (if available): alveolar syndrome, interstitial syndrome.

**Critical COVID-19:** characterized by acute respiratory distress, septic condition and sometimes septic shock. Hypertension was defined as a systolic blood pressure ≥140mmHg, diastolic blood pressure ≥90mmHg, or current use of antihypertensive medication. Diabetes mellitus was defined as a fasting serum glucose ≥126mg/dL, hemoglobin A1c (HbA1c) ≥6.5%, or current use of insulin or anti-diabetic medications. Confirmed cases were defined by the positive findings in reverse-transcriptase- polymerase-chain-reaction (RT-PCR) assay of throat swab specimens [16]. The criteria for hospital discharge were define by absence of fever for at least 3 days, clinical remission of symptoms and two SARS-COV-RNA negative throat swab specimens obtained at least 48 hours after. Conventional treatment: treatment with chloroquine and azithromycin according to the national policy. No conventional treatment: treatment without chloroquine or treatment with chloroquine but interrupted due to side effects. Treatment without chloroquine was recommended to patients with retinopathy, QTc interval ≥500, taking neuroleptics such as citalopram, hydroxyzine etc.

**Treatment according national guideline in Democratic Republic of the Congo:** for mild COVID-19: chloroquine 250mg twice per day and azithromycin 500mg once on day 1 then 250mg daily for 4 days. For moderate COVID-19: same treatment as for the mild form but add anticoagulation at prophylactic dosing. For severe or critical COVID-19: same treatment as in the mild form add anticoagulation at therapeutic dosing. In all 3 cases, symptomatic treatment is recommended: antipyretics if fever, vitamin C 500mg twice daily at all cases, oxygen therapy (nasal catheter or mask) or assisted ventilation according to degree of respiratory distress, antibiotics, rehydration and correction of hydro-electrolytic disorders as appropriate. No use of anti-inflammatory drugs. Mild cases were not admitted but if the patient had comorbities, he could be admitted [17].

### Laboratory procedures

**Confirmation of diagnosis:** oropharyngeal or nasopharyngeal swab were collected from each patient for viral nucleic acid detection of SARS-COV-2 using RT-PCR assay as previously described [18]. The specimens were sent to the National Institute of Biomedical Research.

**RT-PCR:** for each patient, RT- PCR is performed on admission for the confirmatory diagnosis, on day 12 for the control and on day 14 if the control on day 12 was negative. If the check on day 12 remains positive, the check will be done after 7 days.

**Statistical analysis:** statistical analysis was performed with SPSS Statistics Software (version 21; IBM, New York, USA). Continuous variables were expressed as median (IQR) or mean (SD). For continuous variables, the comparisons between the survivor and non-survivor groups were made on a case-by-case basis using student's t-test (variables normally distributed as age, blood oxygen saturation, potassium) or Mann Whitney's test (variables not normally distributed as glycemia, urea, creatinine and CRP). Categorical variables were presented as number (%) and compared using χ2 test or Fisher´s exact test between survivors and non-survivors where appropriate. Kaplan Meier's method described survival from the first day of hospitalization until death (complete data) to the end of the study (censored data). We used the Log-Rank test to compare the survival curves. The Cox regression looked for independent predictors of mortality. The multivariate Cox model was adjusted for the following variables: patients age, sex, civil status, admission procedure, comorbidities, all admission signs, blood oxygen saturation, severity of illness, conventional treatment and biologic variables (glycemia, potassium, urea, creatinine, CRP). A value of p <0.05 was considered as the threshold of statistical significance.

**Ethical considerations:** the data was collected anonymously and confidentially. The privacy and personality of the patients were safeguarded. The three fundamental principles of ethics were respected: respect for the human person, beneficence and justice.

## Results

**Figure 1:**
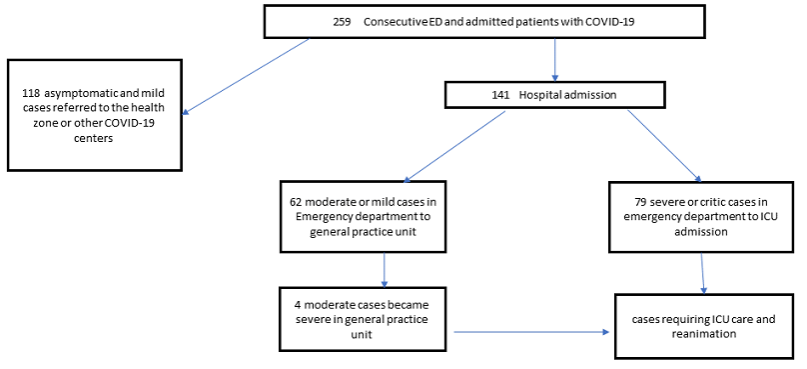
PRISMA diagram of disposition of consecutive patients with COVID-19

**General and biological characteristics of the study population:** of 259 consecutive patients with COVID-19 who were admitted to the hospital between March 23^rd^ and June 15^th^, 141 were hospitalized with COVID-19 followed during the study period (Figure 1), 67.4 % were men (sex ratio 2H: 1F); their average age was 49,6±16.5 years. The majority were married. Nearly half had fever, cough and signs of respiratory distress. Oxygen saturation averaged 84.9+-15.5. Half of patients (56%) were admitted with a severe or a critical illness. Comparing with the survivor, we found that the deceased COVID-19 were significantly old (p <0.001), married (p < 0.001); had antecedent of hypertension (p=0.018) and diabetes mellitus (p=0.044), had dyspnea (p=0.001), low blood oxygen saturation (p <0.001) and signs of respiratory distress (p <0.001) and came with a severe or critical stage of illness (p <0.001) (Table 1). The mean and median values of the biological characteristics of COVID-19 was pathological. When compared to survivors, deceased COVID-19 had significantly higher medians of glycemia and CRP (Table 2).

**Table 1:** general characteristics of 141 patients admitted at the COVID-19 treatment center of the Kinshasa University Hospital, from March 23^rd^ to June 15^th^, 2020

Variables	Total n=141	Survivor n=100	Non-survivor n=41	p
**Age**	**49,6±16.5**	**46,2±16,3**	**57,8±14.2**	**<0.001**
<40 years	38(27,0)	35(35,0)	3(7,3)	
40-59 years	58(41,1)	40(40,0)	18(43,9)	
≥60 years	45(31,9)	25(25,0)	20(48,8)	
**Sex**				0.523
Male	95(67,4)	67(67,0)	28(68,3)	
Female	46(32,6)	33(33,0)	13(31,7)	
**Civil status**				0.001
Married	89(63,1)	58(58,0)	31(75,6)	
Single	41(29,1)	37(37,0)	4(9,8)	
Widower	11(7,8)	5(5,0)	6(14,6)	
**Admission procedure**				0.140
First admission	116(82,3)	85(85,0)	31(75,6)	
Reference	25(17,7)	15(15,0)	10(24,4)	
**Comorbidities**				
Hypertension	33(23,4)	18(18,0)	15(36,6)	0.018
Diabetes mellitus	24(17,0)	13(13,0)	11(26,8)	0.044
Asthma	4(2,8)	4(4,0)	0(0,0)	-
Cardiopathia	6(4,3)	4(4,0)	2(4,9)	0.563
Tuberculosis	1(0,7)	1(1,0)	0(0,0)	-
Pregnancy	5(3,5)	5(5,0)	0(0,0)	-
Obesity	1(0,7)	1(1,0)	0(0,0)	-
**Admission signs**				
Headache	18(12,8)	14(14,0)	4(9,8)	0.352
Coma	1(0,7)	0(0,0)	1(2,4)	-
Fever	77(54,6)	50(50,0)	27(65,9)	0.062
Vomiting	2(1,4)	2(2,0)	0(0,0)	-
Diarrhea	3(2,1)	3(3,0)	0(0,0)	0.354
Abdomen pain	2(1,4)	2(2,0)	0(0,0)	-
Anorexia	3(2,1)	3(3,0)	0(0,0)	-
Chest pain	5(3,5)	4(4,0)	1(2,4)	0.546
Body ache	3(2,1)	3(3,0)	0(0,0)	-
Asthenia	11(7,8)	10(10,0)	1(2,4)	0.116
Cough	84(59,6)	58(58,0)	26(63,4)	0.344
Throat pain	33(23,4)	20(20,0)	13(31,7)	0.103
Anosmia	1(0,7)	1(1,0)	0(0,0)	-
Runny nose	32(22,7)	22(22,0)	10(24,4)	0.459
Dyspnea	93(66,0)	57(57,0)	36(87,8)	<0.001
Bos (%)	84,9±15.5	90,3±10,3	71,2±17.6	<0.001
≤95	95(67,4)	56(56,0)	39(95,1)	
>95	46(32,6)	44(44,0)	2(4,9)	
Signs of respiratory distress	75(53,2)	43(43,0)	32(78,0)	<0.001
**Severity of illness**				**<0.001**
Mild	43(30,5)	42(42,0)	1(2,4)	
Moderate	19(13,5)	16(16,0)	3(7,3)	
Severe or critical	79(56,0)	42(42,0)	37(90,2)	
**Conventional treatment**				**0.146**
No	14(9.9)	13(13.0)	1(2.4)	
Yes	127(90.1)	87(87.0)	40(97.6)	

BOS: blood oxygen saturation; p-value <0.05

**Table 2:** biologic characteristics of patients admitted at the COVID-19 treatment center of the Kinshasa University Hospital, from March 23^rd^ to June 15^th^, 2020

Variables	n	Total	Survivor	Non-survivor	p
Glycemia (mg/dL)	36	109.0 (94.0-203.0)	94.0 (90.0-96.0)	156.0 (109.0-203.0)	0.586
Potassium (mmol/L)	14	3,9±0.6	3,9±0.6	3,8±0.8	0.749
Urea (mg/dL)	30	50.0 (32.0-51.4)	50.0 (48.0-55.0)	41.7 (32.0-51.4)	0.709
Creatinin (mg/dL)	33	1.4 (1.0-1.6)	1.6 (1.1-1.9)	1.2 (1.0-1.4)	0.454
CRP (mg/L)	15	96.0 (36.0-450.0)	36.0 (33.1-39.2)	273.0 (96.0-450.0)	0.581

Data are presented as n (the total number of non-missing values in each category), median (interquartile range) or mean ± SD. CRP: C reactive protein. p-value <0.05

**Mortality rate for COVID-19 at Kinshasa University Hospital:** of the 41 deceased, 10 (24%) deceased within 2 hours in the transition zone and 70% deceased within 24 hours of admission. Thirty-one patients were treated intra-hospital. Mortality of hospital was 16% for all patients at KUH and 29% for hospitalized patients.

**Figure 2:**
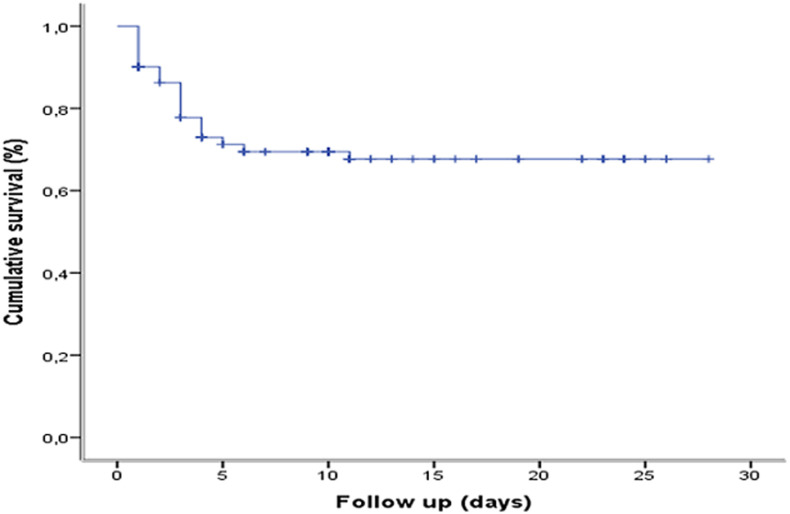
overall survival of COVID-19 at the COVID-19 treatment center of the Kinshasa University Hospital, from March 23^rd^ to June 15^th^, 2020

**Survival of COVID-19 patients at Kinshasa University Hospital:** overall survival of COVID-19. The probability of survival for COVID-19 patients at the start of follow-up was 100%, 90%, 78.7%, 73.0%, 71.6% and 70.9%, respectively, at 1 day, 2 days, 3 days, 5 days, 10 days and 30 days. The median survival of COVID-19 patients was 9 (5-10) days (Figure 2).

**Figure 3:**
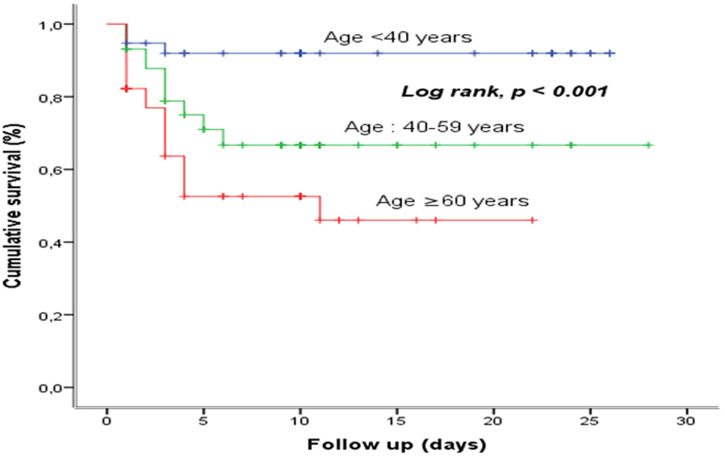
survival based on age group at the COVID-19 treatment center of the Kinshasa University Hospital, from March 23^rd^ to June 15^th^, 2020

**Survival based on age group:** young tended to have better survival compared to old, with a statistically significant difference (p <0.001) (Figure 3).

**Figure 4:**
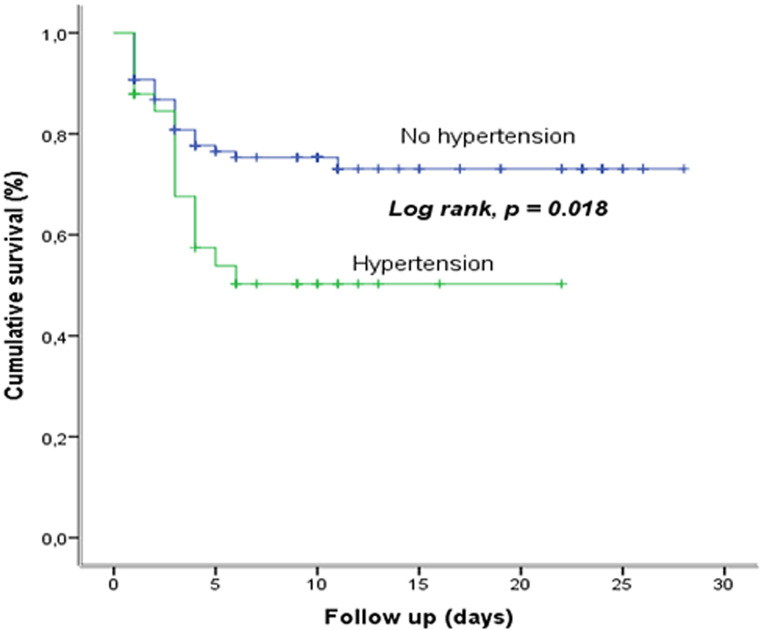
survival based on hypertension at the COVID-19 treatment center of the Kinshasa University Hospital, from March 23^rd^ to June 15^th^, 2020

**Survival based on hypertension:** the comparison of the survival curves of COVID-19, according to the presence or not of hypertension shows a statistically significant difference (p=0.018); the presence of hypertension reduces the survival (Figure 4).

**Figure 5:**
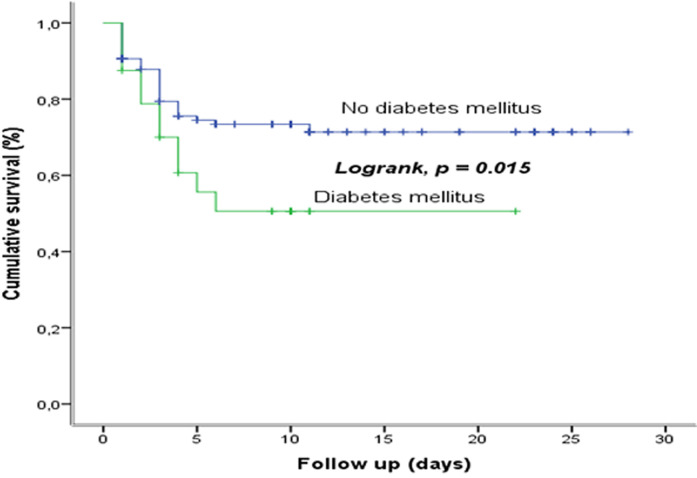
survival based on diabetes mellitus at the COVID-19 treatment center of the Kinshasa University Hospital, from March 23^rd^ to June 15^th^, 2020

**Survival based on diabetes mellitus:** the comparison of the survival curves of COVID-19, according to the presence or not of DM shows a statistically significant difference (p=0.015); the presence of DM reduces the survival (Figure 5).

**Figure 6:**
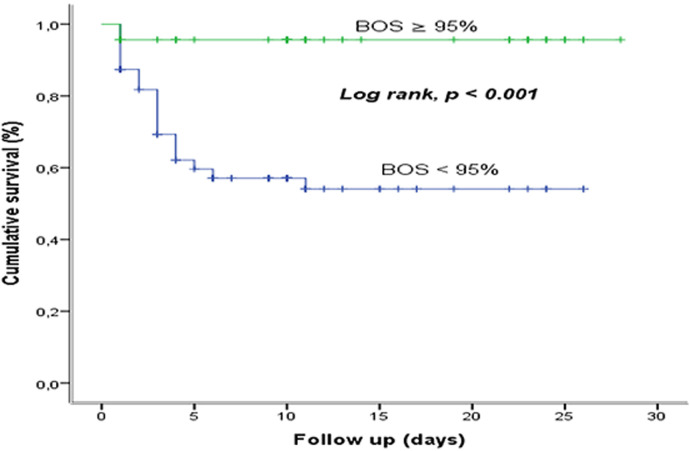
survival based on blood oxygen saturation at the COVID-19 treatment center of the Kinshasa University Hospital, from March 23^rd^ to June 15^th^, 2020

**Survival based on blood oxygen saturation:** the comparison of the survival curves of COVID-19, according to the low or high BOS shows a statistically significant difference (p <0.001). The low BOS reduces the survival (Figure 6).

**Figure 7:**
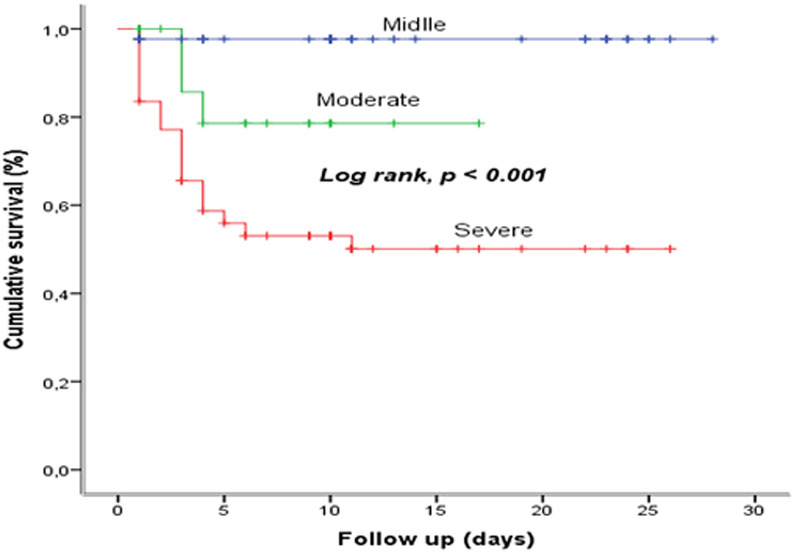
survival based on severity at the COVID-19 treatment center of the Kinshasa University Hospital, from March 23^rd^ to June 15^th^, 2020

**Survival based on severity:** the comparison of the survival curves of COVID-19, according to the severity of illness at admission shows a statistically significant difference (p <0.001). The severe or critical COVID-19 reduces the survival (Figure 7).

**Predictors of mortality in COVID-19 patients:** in multivariate analysis, age between 40 and 59 years (aHR: 4.07; 95% CI: 1.16-8.30), age at least 60 years (aHR: 6.65; 95% CI: 1.48-8.88), severe COVID-19 (aHR: 14.05; 95% CI: 6.3-15.67) and dyspnea as sign at admission (aHR: 5.67; 95% CI: 1.46-21.98), were independently associated with mortality (Table 3).

**Table 3:** predictors of mortality in COVID-19 infected patients admitted at the COVID-19 treatment center of the Kinshasa University Hospital, from March 23^rd^ to June 15^th^, 2020

Independant factors	β	S.E	Wald	Ajusted HR (95%CI)	p-value
**Age**					
<40 years				1	
40-59 years	1.40	0.64	4.793	4.07 (1.16-8.30)	0.029
≥60 years	1.89	0.66	8.350	6.65 (1.84-10.07)	0.004
**Admission procedure**					
First admission				1	
Reference	0.21	0.44	.231	1.23 (0.52-2.91)	0.631
**Hypertension**					
No				1	
Yes	0.07	0.38	.033	1.07 (0.51-2.26)	0.857
**DM**					
No				1	
Yes	0.27	0.40	.440	1.31 (0.59-2.88)	0.507
**BOS**					
≥92%				1	
<95%	0.41	1.06	.149	1.51 (0.19-12.04)	0.700
**Severity**					
Mild				1	
Moderate	3.52	1.66	4.513	3.79 (1.31-9.51)	0.034
Severe	4.98	1.60	9.710	14.05 (6.37-15.67)	0.002
**Fever**					
No				1	
Yes	0.29	0.40	.519	1.33 (0.61-2.90)	0.471
**Headache**					
No				1	
Yes	0.39	0.55	.493	1.47 (0.50-4.35)	0.483
**Runny nose**					
No				1	
Yes	0.41	0.43	.914	1.50 (0.65-3.46)	0.339
**Dyspnea**					
No				1	
Yes	1.73	0.69	6.289	5.67 (1.46-21.98)	0.012

p-value <0.05; β: coefficient of regression; Hr: hazard ratio; SE: standard error

## Discussion

The objectives of this study were to measure the mortality rate of patients who died from COVID-19, assess survival and identify predictive factors of mortality from COVID-19 patients. The mortality rate in infected COVID-19 patients was 29% during the study period. Ten patients deceased within the first 2 hours. Survival was decreased with the presence of hypertension, diabetes mellitus, low blood oxygen saturation, severe and moderate stage disease. Older age, severe COVID-19 and dyspnea as sign in admission were independently associated and significantly at risk of death. The mortality rate for hospitalized patients with COVID-19 was 29% during the study period. The result was similar than F Zou *et al*. with 28% mortality rate in China [10]. The literature found that the overall mortality rate of COVID-19 is much lower than for severe acute respiratory syndrome (10%) and Middle East respiratory syndrome (30%) [19,20]. However, COVID-19 has ultimately proven more deadly as it has spread to many more people globally than did the others, owing to rapid to person transmission and atypical symptoms at an early stage in certain patients [21,22]. Although in the brief report on patients hospitalized until April, Mandina *et al*. reported a high mortality rate of 10% [23]. Here, we reported a very high mortality rate for COVID-19, which is higher than in recent reports. This might be due to the fact that this hospital being a tertiary care public hospital, all serious cases were referred by other hospitals. A large proportion of severely or critically ill patients were admitted at Kinshasa University Hospital. Out of all patients, 56% were severe and critical. The late consultation or reference had also induced died. Some patients had already come in dead.

Survival decreases with age, patients over 65 years of age had poorer survival than those with 0-59 years of age or less than 40 years of age. Frailty, which was labeled as “the most problematic expression of population ageing”, is often confused with disability or co-morbidity. But frailty is assuming separate distinguished features, which highlight the presence of a well-defined phenotype that characterizes elders. Many elders have multiorgan dysfunction, with frailty representing a “unifying notion in the care of elderly patients that directs attention away from organ-specific diagnoses”. The concept of frailty is now universally defined as an individual´s state of increased vulnerability and susceptibility to adverse health outcomes or death when exposed to a stress or in this case the pulmonary diseases arising from SARS-COV-2 infection in the presence of an age-dependent excess of ROS and reactive nitrogen species (RNS) [24]. In the present study, survival was decreased with the presence of hypertension, diabetes mellitus, low blood oxygen saturation, severe and moderate stage disease. Hypertension may also represent a proxy for the presence of other cardiovascular risk factors such as diabetes, hypertension mediated target organ damage or cardiovascular complications, all of which show an increasing prevalence with age [25]. Patients with diabetes are more susceptible to be infected by virus, bacteria and fungus than individual without diabetes owing to relatively lower immune function. As a result, these patients might be at an increased risk of SARS-COV-2 infection and consequently poor prognosis. We found that COVID-19 patients with diabetes were more likely to develop severely or critically ill subtypes, including more complications with ARDS, acute cardiac injury, resulting in receiving more antibiotic therapy and mechanical ventilation [26-28].

Severe pneumonia can cause significant gas exchange disturbances and lead to hypoxemia. Hypoxia reduces the energy production required for cell metabolism and increases the body's anaerobic digestion. Acidosis and oxygen free radicals accumulated in the cell destroy the phospholipid layer of the cell membrane. As hypoxia continues, the intracellular calcium ion concentration increases significantly, leading to a series of cell damage processes, including apoptosis [29]. At the same time, hypoxia can also induce inflammatory reactions, such as the infiltration of inflammatory cells and the release of cytokines, leading to further tissue ischemia and may even cause myocardial infarction [30]. In this study, the predictors are advanced age, severe and moderate stages and the presence of dyspnea as a sign of admission. The current study confirmed that increased age was associated with death in patients with COVID-19. In the last outbreak, older age has been reported as an important independent predictor of mortality in SARS and MERS [31,32]. When they compare older to young macaques inoculated with SARS-COV-19 in previous studies, the authors found that older macaques had stronger host innate responses to virus infection than younger adults, with an increase in differential expression of genes associated with inflammation, whereas expression of type I interferon beta was reduced [33].

The age-dependent defects in T-cell and B-cell function and the excess production of type 2 cytokines could lead to a deficiency in control of viral replication and more prolonged proinflammatory responses, potentially leading to poor outcome [34]. According to severity, the severe illness occurred in 56% of the patients after admission to a hospital. The frequency in the study of Guan *et al*. was lower (12.5%) and they found that patients with severe disease were older than those with non-severe disease, the presence of any coexisting illness was more common among patients with severe disease than among those with non-severe disease (38.7% vs 21.0%). However, the exposure history between the two groups of disease severity was similar [35]. Dyspnea is a general sign in COVID-19 and is a reason to consult for severe cases. It is found mainly in patients with COVID-19 pneumonia, COVID-19 associated pulmonary embolism and also reflects other COVID-19 associated heart disease. Soares *et al*. found also dyspnea as risk factor of mortality of hospitalized COVID-19 patients in Brazil [11].

**Limitation:** our study has some limitations. Firstly, due to the retrospective study design, not all laboratory tests were done in patients, including d dimer, IL-6, troponin, lactate dehydrogenase and serum ferritin. Therefore, their role might be underestimated in predicting in-hospital death. Secondly, patients were sometimes transferred late in their illness, this situation overestimate the mortality rate. Thirdly, the median length of hospital admission before death was about three days, information on the dynamic changes in laboratory variables in deceased patients was lacking and the data collected for each patient on admission may have been from different disease stages.

## Conclusion

Older age, severe or critical COVID-19 and dyspnea on admission were potential predictors of fatality in patients with COVID-19. These predictors may help clinicians identify patients with a poor prognosis in admission.

### What is known about this topic

- Age, sex and co-morbidities such as hypertension, diabetes, cardiovascular disease, chronic respiratory disease and cancer are risk factors of COVID-19 mortality;
- D dimer, IL-6, troponin, lactate dehydrogenase and serum ferritin are biological parameter associated to COVID-19 mortality;
- The mortality rate for COVID-19 is high in patients in intensive care unit or reanimation.

### What this study adds

- The overall mortality rate in infected COVID-19 patients was 29% in the COVID-19 treatment center of Kinshasa University Hospital;
- Survival decreased with the presence of hypertension, diabetes mellitus, low blood oxygen saturation (BOS), severe and moderate stage disease;
- Older age, severe or critical COVID-19 and dyspnea on admission were potential predictors of fatality in patients with COVID-19.
